# Conceptual Framework for African American Kinship Caregiver’s Susceptibility to Alzheimer’s Disease

**DOI:** 10.3390/healthcare12232379

**Published:** 2024-11-27

**Authors:** Tyreasa Washington, Sheryl Coley, Joan Blakey, Kenya Downing, Quiana Lewis Wallace, Sue Levkoff, Benjamin Cook

**Affiliations:** 1 Child Trends, Rockville, MD 20852, USA; kdowning@childtrends.org (K.D.); qlewiswallace@childtrends.org (Q.L.W.); 2 Department of Social Work, University of North Carolina at Greensboro, Greensboro, NC 27412, USA; slcoley@uncg.edu; 3 School of Social Work, University of Minnesota—Twin Cities, St Paul, MN 55108, USA; blak0014@umn.edu; 4 College of Social Work, University of South Carolina, Columbia, SC 29208, USA; slevkoff@mailbox.sc.edu; 5 Department of Psychiatry, Harvard Medical School, Boston, MA 02115, USA; bcook@cha.harvard.edu

**Keywords:** black, child welfare, grandparents raising grandchildren, dementia, health disparities

## Abstract

Kinship caregivers (e.g., grandparents raising grandchildren) have been increasing over the last several decades. Approximately 3.5 million grandparents and other relatives are the primary caregivers for their related children, and African Americans are more likely to be kinship caregivers than persons from other groups. Kinship caregivers face unique challenges, such as parenting for uncertain periods of time and often with insufficient financial resources and support, placing them at significant risk of stress. Given the findings linking chronic stress to Alzheimer’s disease (AD), there is a need for research to identify possible stressors and mitigate risks for outcomes such as AD among kin caregivers. Additionally, research indicates that African Americans (AAs) experience unusually high levels of stress due to factors often associated with structural racism, and they are disproportionately affected by cardiovascular disease (CVD), which is often a consequence of stress and another risk factor for AD. Regrettably, AA kin caregivers often incur a host of negative stress-related outcomes, including poor physical and mental health. Thus, there is an urgent need for research to identify modifiable risk factors for both stress and CVD to potentially mitigate the onset of AD in this population. The purpose of this paper is to provide a conceptual framework to examine the links between African Americans who commit to the unselfish act of providing kinship caregiving and their susceptibility to AD. Future research should investigate modifiable mechanisms to reduce the risks of AD in African American caregivers.

## 1. Kinship Care

When biological parents are unable or unwilling to care for their children and relatives, family members who take on the role of the primary caregivers are known as “kinship caregivers” [1]. Two primary types of kinship care exist: formal and informal. Formal kinship care arrangements are supervised by child welfare agencies. Informal kinship care arrangements are not under the supervision of the child welfare system; therefore, the state does not have legal custody of the children. Approximately 30 to 35 percent of children in kinship care reside in formal kinship care families, and around 70 percent of kinship care families are informal [1]. Other research reports that for every child in formal kinship care arrangements, there are approximately twenty children in informal arrangements [2].

Kinship care families are one of the fastest-growing family types. There are approximately 2.5 million grandparents acting as primary caregivers for their grandchildren, and 1 million other relatives, such as aunts or uncles, filling this role [3]. The ages of kin caregivers are sixty and beyond for about 51% of all grandparents who are responsible for their grandchildren, while about 49% are aged thirty to fifty-nine. In general, kinship caregivers face unique challenges, such as unexpectedly becoming a primary caregiver to child(ren), often with limited financial resources [4], placing them at significant risk for stress [5,6]. Also, African Americans are overrepresented among persons caring for related children [3,7], and they often display high stress levels [8,9]. Because studies have indicated links between chronic stress and Alzheimer’s disease (AD) [10,11], the high stress levels of a person caring for related children are alarming. Additionally, since stress is also related to cardiovascular diseases [12,13], and research indicates that both stress and CVD may contribute to the progression of AD [11,14], this is another reason to be alarmed. Furthermore, given that African Americans are overrepresented in kinship care, as well as highly stressful situations associated with structural racism, and disproportionally affected by CVD and AD, then African American kinship caregivers should be considered a vulnerable group for developing AD [15,16,17,18]. The purpose of this paper is to provide a conceptual framework for the links between African American kin caregivers and their susceptibility to AD. Additionally, it will highlight the need for research to identify modifiable risk factors for stress and CVD for this group to potentially mitigate the onset of AD in AA kinship caregivers.

## 2. Methods

For this conceptual paper, the research team developed the database and protocol search strategies in consultation with a university librarian. We searched the following databases: PubMed, PsycINFO, SocIndex, Social Service Abstracts, and Sociological Abstracts. These databases were chosen based on the objectives of the paper and recommendations from the librarian, including disciplinary topic-specific databases that contain relevant health journals.

We conducted searches using combinations of the following keywords and phrases: “African American”, “Black”, “kinship care”, “Alzheimer’s”, “Alzheimer’s and dementia”, “pathologies of CVD and AD”, and “relationships among stress, CVD, and AD”. Additionally, we utilized thesaurus tools within the databases to identify controlled terms for these keywords when appropriate.

To ensure comprehensive coverage, we employed further methods to locate studies that may not have been identified through database searches. This included reverse searching, where we examined citations and references of relevant studies, as well as reviewing National Institute of Health abstracts and other materials focused on Alzheimer’s disease and cardiovascular disease.

## 3. African Americans and Kinship Care

African Americans are overrepresented among persons caring for related children [3,7] and this has been consistently confirmed over the past decades [7,19,20]. For example, Amorim and colleagues’ [7] research indicates that African American children are almost twice as likely to ever live with their grandparents as the primary caregiver compared to Hispanic children, three times as likely as White children, and six times as likely as Asian children. Additionally, research indicates that African American caregivers are almost twice as likely as the overall population to participate in kinship care, most of which is informal [20].

Scholars and researchers suggest that the overrepresentation of African Americans in kinship care is an adaptation and strength used by the African American community to address systemic racial oppression that dates back to enslavement [20,21,22]. For example, when enslaved children were separated from their birth parents, other enslaved persons took care of those children. During the Great Migration, which was the mass movement of African Americans from the South to other regions of the United States, many birth parents left their children in the South with relatives while they went in search of education and economic opportunities. As a result, this also contributed to the disproportionate number of Black children in kinship care [22,23,24]. Historically, kinship care has been a strength of the African American community, and in modern times, it is often used to address factors such as parental substance use, incarceration, and mental illness [8]. Many of these factors are associated with structural racism [25,26,27,28].

Considering the unselfish acts of African Americans who provide kinship care and the health risks that come along with being a caregiver, it is imperative to identify means to promote their overall health and well-being. Children placed in kinship care have better mental health, behavioral, and academic outcomes than children placed in traditional foster care [29,30,31,32]. Thus, child welfare stakeholders, as well as society in general, should be invested in the health of caregivers since it benefits children who do not live with at least one birth or adoptive parent and lessens the need for the involvement of child welfare systems.

## 4. Stress 

Stress describes the effect of stimuli on an individual’s psychological, emotional, and physical sense of well-being [33,34,35]. At times it can be “good stress”, but almost always, when stress is referred to, it is about “bad stress”, which is the type of stress this paper will focus on. Stress commonly results from financial, systemic, and interpersonal strains, and the effects of it are called the stress response system. This system is a mechanism providing an evolutionary advantage in response to a threat, which prepares the body to fight or flee [36]. Historically, this response has provided a temporary escape from threats. When the threat is no longer present, an “all-clear” message is sent to the body, signaling it can shift back to homeostasis or a relaxed state [37]. However, many facets of modern American life have contributed to the prevalence of chronic stress, in which the brain is incapable of settling down to a relaxed baseline. What is often seen in chronic stress is that the baseline has shifted, so a lower level of stress response becomes the new baseline rather than the absence of stress itself. If left unaddressed and untreated, chronic stress harms the biological system, affecting the cardiovascular system, learning and memory, and psychological well-being (among others). Specifically, over time, chronic stress may accelerate biological aging, lower the immune system response, and contribute to chronic disease [38,39]. Stress can also increase susceptibility to dementia and Alzheimer’s disease, a chronic condition in which a person’s cognitive function has been affected, with symptoms most associated with thinking and memory [39,40,41,42].

## 5. Stress Among African Americans

Marginalized groups are particularly susceptible to chronic stress. Specifically, research indicates that African Americans have higher stress levels than Whites [43,44]. Additionally, nearly 12 percent of African American adults report serious psychological distress [45]; suicide ranks as the third leading cause of death among young African Americans, and these deaths by suicide appear to be linked to stress [46]. Importantly, chronic stressors for African Americans are associated with factors related to structural racism. Williams and colleagues [47] report that stressful life experiences are associated with social structures and systems, which significantly affect the types and quantities of stress African Americans encounter [47]. An example includes a recent study that found that African Americans’ tax returns are audited by the federal government up to five times more than their White peers [48]. Additionally, prosecutors are more likely to charge African American individuals with criminal charges that carry more severe sentences compared to White individuals in similar cases [49].

Furthermore, the disproportional negative outcomes that people of color experience in America, particularly African Americans, undoubtedly affect this population’s stress levels and other unfavorable health-related outcomes. For instance, African Americans are often victims of unfair sentencing that is often linked to systematic racism, and a consequence of this is them being nearly six times more likely than White adults to be imprisoned [50]. Similar disparities persist in the ways these families are monitored by the child welfare system. Over half (53%) of African American children, nearly twice as many as White children (28%), are investigated by Child Protective Services at some point during childhood [51].

The exposure to chronic stress among African Americans is often linked to structural racism and undoubtedly contributes to their disproportionate physical and mental health deterioration [28,47,52,53].

## 6. Stress and African American Kinship Care

The research above has confirmed that almost all kinship caregivers have ample reasons to have elevated stress levels, as well as African Americans in the general population. However, African American caregivers most likely face challenges to greater degrees than other caregivers, which could increase their stress levels. For example, African American kinship caregivers may experience additional stressors related to racism throughout their lifetimes, which may further heighten their stress levels. Smith-Ruiz and Jason [54] observed through their mixed-method comparison between African American and White caregivers that the need for support and difficulty of obtaining help for African American caregivers to handle children’s academic challenges were almost twice the magnitude of White caregivers. 

As a whole, financial strain can impact grandparents and other relatives who provide kinship care, and contribute to negative outcomes [5,6,55]. However, the relationship between financial instability and poorer health among African American kinship caregivers needs more investigation, given that African Americans continuously face greater financial burdens when compared with other racial and ethnic groups. In exploring relationships between financial burden and stress, one study showed that among a sample of kin caregivers, over 90 percent were African American, and found that the lack of financial and material resources worsens kinship caregiver stress [8]. In a recent qualitative study, Woods [56] found that financial difficulties were reported as stressors among rural African American grandmothers who provide kinship care and who experience chronic health issues. Additionally, the results from a very recent study by Washington and Despard [4] produced consistent findings with their examination of data from their mixed-method research involving African American informal kinship caregivers and data from a national dataset. Their research revealed that African American kinship caregivers were more likely to have insufficient financial and family resources when compared with other US families, and the financial burdens that result can increase the risks of caregivers’ health problems. 

A recent literature review noted the limited research on kinship caregivers who had diverse racial and ethnic identities, as well as other heterogeneous characteristics [57]. Further, even though some research exists about stress among African Americans, less literature is available about kin caregivers and stress, and the literature concerning the long-term consequences of stress on this population is very scant. 

## 7. Stress and Alzheimer’s Disease

Scientific advances have linked stress to Alzheimer’s disease [11,42,58,59]. Historically, it has been found that ongoing, chronic stress is a specific type of stress that leads to the development of neural degeneration, pathologic alterations, and the progression of Alzheimer’s disease and symptom severity [59,60]. For example, Alkadhi and Tran [61] examined whether chronic psychosocial stress could hasten the appearance of Alzheimer’s disease symptoms, including changes in basal levels of cognition-related signaling molecules, in subjects at risk for the disease. The study’s findings suggested that changes in the basal levels of signaling molecules may be responsible for impaired learning, memory, and long-term potentiation based on a rat model. Other research involving animal models conveyed that stress could perpetuate the elevation of cortisol levels, which are commonly found in persons with AD [41]. Stress can subsequently lead to hippocampus degeneration, affecting the brain’s immune system, exacerbating neuroinflammation, and leading to the progression of AD [41,42]. In addition to chronic stress being a type of stress risk factor, post-traumatic stress disorder (PTSD) is another type of stress that researchers indicate is related to dementia and AD. For example, in studies of aging individuals, including veterans, who suffer from neurodegenerative diseases, it was observed that individuals diagnosed with PTSD were up to twice as likely to be diagnosed with dementia. In fact, stress is known to worsen several disorders, including Alzheimer’s disease [59,61,62].

The exact causative pathways of Alzheimer’s disease are still under investigation, but our current understanding of the condition has identified that APOE-ε4 is the strongest genetic risk factor for AD. This gene is associated with an increase in the levels of amyloid deposition [63]. Research indicates that APOE-ε4 is the strongest genetic risk factor for AD. This gene is associated with an increase in the levels of amyloid deposition, as well as early age of onset [63]. However, the majority of studies conducted on AD have been with non-Hispanic Whites, despite African Americans having 2–3 times higher rates of AD than non-Hispanic Whites [64,65]. In 2023, a study was conducted by Groechel and colleagues that exclusively examined AD biomarker abnormalities in 85 African Americans who were placed in three groups: cognitively normal, mild cognitive impairment, or dementia. Researchers examined these individuals’ demographics, apolipoprotein E (APOE) ε4, cerebrospinal fluid (CSF) Aβ1-42, CSF total tau (t-tau), CSF phosphorylated tau (p-tau), 3T magnetic resonance imaging (MRI), and measures of cognition and function. Their findings confirmed greater AD biomarker abnormalities among the African American clinical groups in this sample. 

It is essential to acknowledge that genetic factors are largely immutable, particularly in the short term. Therefore, our research team will concentrate on behavioral risk factors for Alzheimer’s disease that can be modified, aiming to achieve the most significant and immediate benefits for this population.

## 8. Cardiovascular Disease

Cardiovascular disease is the leading cause of mortality and morbidity in the US. Psychological stressors are a type of stress that is associated with CVD [66]. This form of stress encompasses the mental and emotional strain that arises when individuals perceive a situation as challenging or threatening, such as experiences of social isolation or work-related challenges. Another primary behavioral risk factor for CVD is behaviors that include unhealthy diet patterns, lack of physical activity, smoking and tobacco use, and alcohol abuse [12,33]. The Centers for Disease Control estimates that CVD contributes to more than 800,000 deaths per year [33]. It is the leading cause of death in the United States [67]. The prevalence of CVD in the population is 48%. However, there are disparities by race. Nearly 60% of non-Hispanic Black adults report having CVD, which represents the highest prevalence of CVD by race [67]. A study conducted by Mohebi and colleagues modeled the future prevalence of CVD and estimated that by 2060, the prevalence of CVD would decrease among White populations while there would be significant increases among racial minorities, especially African Americans [12,62,63].

## 9. Cardiovascular Disease Among African Americans

African Americans continue to have a higher prevalence of CVD compared to non-Hispanic Whites [17,18,68]. Recent National Health and Nutrition Examination Survey (NHANES) data indicated that among Americans over 20 years old, the prevalence of CVD remains the highest among African American women, followed by African American men [67]. CVD is the leading cause of death for Black women and significantly contributes to the life expectancy gap between Black and White women, which stands at 78.4 years and 81.4 years, respectively [69]. Hypertension, a key risk factor for CVD, is nearly twice as prevalent among Black women compared to White women. Researchers have also identified connections between these social determinants and health outcomes, noting that racism is associated with CVD risk factors such as obesity [65]. Residential segregation, in particular, has been found to be a contributing factor to increased CVD incidence among African Americans in contrast to decreased CVD prevalence among non-Hispanic Whites [70]. Additionally, these disparities in health are associated with social and economic factors such as inequities in access to quality healthcare services, economic stability, safe neighborhoods, and stable housing [18,68].

## 10. CVD Among African American Kinship Caregivers

Challenges related to caregivers’ physical health are commonly identified among kinship caregivers, particularly those of lower socioeconomic status [66,67,68] and African American kinship caregivers [71,72,73] African American grandparents, in particular, face increased health challenges when compared with caregivers of other races, including CVD and related risk factors. In comparing data from 13,705 caregivers from the Health and Retirement study, Choi [71] found that significantly more African American grandparent caregivers had heart disease and other chronic conditions than Whites. Additionally, African American grandparents appear more vulnerable than other types of family caregivers. A previous comparison of African American solo parents and grandparent caregivers using data from the Behavior Risk Factor Surveillance Survey (BRFSS) found African American grandparents had CVD at a rate six times higher than that of parents [74].

## 11. CVD and AD

CVD has been linked with stress and Alzheimer’s disease [70]. The relationship between chronic stress and CVD is well-understood and well-represented in the literature [75]. Further, CVD is a common condition with a variety of comorbidities. Individuals with CVD and diabetes have been identified as being at higher risk for developing AD [76,77]. Additionally, the renowned longitudinal CAIDE study (Cardiovascular Risk Factors, Aging, and Incidence of Dementia), which examines the connections between social, lifestyle, and cardiovascular risk factors and dementia, found that dementia was predicted by CVD factors and obesity [78]. In short, over time, stress-related hormones and behaviors (e.g., smoking) can contribute to cardiovascular conditions like high blood pressure, hardening of the arteries, and insulin resistance. These issues can, in turn, lead to changes in the brain that are involved in the onset and worsening of Alzheimer’s disease.

## 12. Stress, CVD, and AD: Gaps in Knowledge

To date, research has revealed important associations between stress, CVD, and AD, which are important to consider for African American kinship caregivers. Stress is well-established to increase cardiovascular problems such as diabetes, hypertension, and stroke [13,79,80]. Additionally, stress and CVD are associated with AD. Recent research continues to increase our awareness that African Americans are disproportionately more likely to have AD and other related dementias [15,16]. African American kinship caregivers can be particularly vulnerable to AD because of the ongoing disparities, with African Americans having higher rates of CVD and other risk factors. Their overrepresentation in kinship care and experiences with chronic stress related to caring for children can put these caregivers more at risk. Taking into account the chronic stress faced by African American kinship caregivers, and the fact that African Americans experience AD at a rate approximately 2–3 times higher than the general population, then it is likely that this population (i.e., African American kinship caregivers) is vulnerable to AD [65]. However, despite these well-known associations and risks, an ongoing gap in the literature on AD among African Americans in general still presents a barrier to understanding these causal pathways specifically among African American kinship caregivers. Furthermore, there is limited research available concentrated on African American caregivers of older adults and persons with AD, and very few studies focused on African American kinship caregivers of children. As a result, there are no known existing interventions to reduce stress to mitigate risk for AD, specifically among African American kinship caregivers of children. 

**Figure 1:**
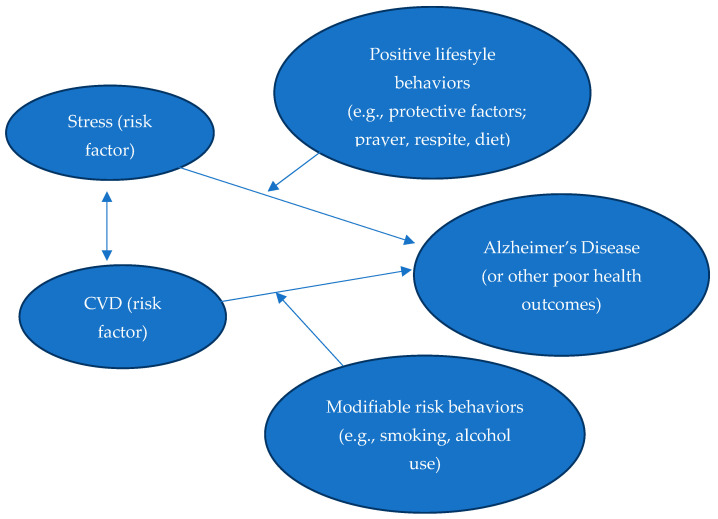
Modifiable Risks for Alzheimer’s Conceptual Framework.

Promoting the health and well-being of African American kinship caregivers must involve reducing health risks, including risks related to stress and CVD, to subsequently reduce their risk for developing AD. Figure 1 below illustrates the theorized pathways between stress, CVD, and AD for African American kinship caregivers. Understanding each of these constructs in light of the unique circumstances of African American caregivers is a critical step for developing culturally sensitive interventions for reducing AD risk in this population. 

## 13. Implications for Practice and Research

Child welfare agencies, practitioners, and other stakeholders should prioritize the health of caregivers for various reasons, particularly in light of increasing practices and policies aimed at diverting children from the child welfare system to kinship. It is essential to provide support to caregivers to ensure that their caregiving responsibilities do not adversely affect their current and future health and well-being [4,81]. We recommend that human service workers coordinate support for kinship caregivers, including stable and secure housing, respite care for children, and assistance with technology, to enhance caregivers’ ability to function effectively in society. Additionally, aiding caregivers in navigating the school system and accessing resources like tutoring services for children would be beneficial. Many of these recommendations could not only reduce stress and the associated risk for cardiovascular disease (CVD) but also promote healthy practices that prevent CVD. For instance, providing respite care could give caregivers time to exercise, and skills learned through technology could help them find information on preventive behaviors for CVD.

We want to emphasize that the supports we recommend above for kinship caregivers can inform future interventions. For example, peer support groups have been found to be a successful intervention [82]; thus, a possible intervention would involve creating support groups specifically for kinship caregivers who share similar experiences and certain demographic characteristics. During these support groups, researchers could guide group leaders to facilitate discussions about what caregivers feel they would need in place to access respite services. The insights gathered from these discussions could then be used by researchers to design and test interventions that address caregivers’ suggestions to improve access to respite services. Another potential intervention would be to provide caregivers with a class on technology, which would allow researchers to assess whether the class enabled them to more easily complete tasks such as checking medical records, scheduling medical appointments, online banking, or using social applications.

Researchers focused on reducing the risk of Alzheimer’s disease (AD) among African Americans should extend their investigations to African American kinship caregivers, who are particularly vulnerable to this disease. The primary aim of our paper is to highlight gaps in the existing research on the interrelationship of factors; specifically, how stress and CVD increase the risk of AD for these caregivers. As a result, we do not delve deeply into potential interventions; instead, we provide justifications for others to develop these interventions. This paper serves as a robust framework for researchers to conduct cross-sectional, longitudinal, and intervention studies that benefit this unique population. 

## 14. Conclusions

In closing, African American kinship caregivers are at risk for developing Alzheimer’s disease due to a variety of interrelated factors. These caregivers are particularly vulnerable because of chronic stress, often arising from their caregiving roles and structural racial inequities, such as inadequate housing, along with the disparities in CVD and AD among African Americans. Despite these well-known associations, there are few, if any, existing prevention strategies and interventions aimed at mitigating AD risk among African American kinship caregivers that take into account their cultural and lived experiences. This conceptual paper represents a first step in identifying the links between African Americans who engage in the selfless act of kinship caregiving and their susceptibility to AD, as well as providing a framework for research to reduce their risk for AD.

## Data Availability

Data are contained within the article.
